# Editorial: Improving disaster health outcomes and resilience through rapid research: Implications for public health policy and practice

**DOI:** 10.3389/fpubh.2022.989573

**Published:** 2022-08-02

**Authors:** Stephanie Montesanti, Iain Walker, Arthur W. H. Chan

**Affiliations:** ^1^School of Public Health, University of Alberta, Edmonton, AB, Canada; ^2^Centre for Healthy Communities, School of Public Health, University of Alberta, Edmonton, AB, Canada; ^3^Research School of Psychology, Australian National University, Canberra, ACT, Australia; ^4^Chemical Engineering and Applied Chemistry, University of Toronto, Toronto, ON, Canada

**Keywords:** disaster research, rapid research, health, mental health, public health policy, research impact

## Rapid research during and following a disaster

The need for researchers to respond to disasters becomes more vital as natural disasters, outbreaks, and pandemics become more frequent and severe across the globe, with devastating, extensive, and prolonged effects on some people's physical and mental health, social and personal relationships, and welfare (1–3). The increasing frequency and severity of natural disasters is exacerbating their cascading and compounding effects. Disaster research helps us to better understand population needs in terms of coping, stress, resiliency, the ability of organizations to deliver urgent services and supports, and the immediate and long-term effects of disasters on the health and wellbeing of residents, communities' and first responders. The findings from disaster research also demonstrate important lessons that can inform current and future policy decisions related to disaster or emergency mitigation, prevention, recovery, and community resiliency. Governments and healthcare organizations across the globe have relied on rapid research efforts to quickly understand the immediate health and social effects from disasters, and to inform prevention, mitigation, and recovery strategies (4–6). Examples of government-funded rapid research include the Ebola, SARS, and Zika Virus outbreaks, major natural disasters including the 2016 Horse River Wildfire in Alberta, Canada and the 2019-2020 bushfires in Australia, and more recently the global COVID-19 pandemic. The current COVID-19 pandemic has seen the scientific community rapidly conduct research to improve our understanding of the virus and its wider societal impacts, and to provide the evidence-base for governments to make decisions (4). Enabling rapid research during and after a disaster allows for the real-time collection and analysis of data essential to an effective evidence-informed response.

Gaillard and Gomez coined the term “research gold rush” to describe the post-disaster rush to identify and understand the immediate effects after high profile disasters (7). Although responses to disaster events typically use the best available science for quickly gathering information to support rapid delivery of services and interventions to communities and populations affected, additional research, done during and after the response itself, is often essential to address pressing knowledge gaps presented by disasters (such as longer-term effects on a population and health system responsiveness to address urgent population health needs) and to ensure that they are addressed by the time another similar disaster happens (8, 9).

In this Research Topic titled, *Improving Disaster Health Outcomes and Resilience through Rapid Research: Implications for Public Health Policy and Practice*, scholars explore research conducted from rapid funding opportunities to address urgent disasters across geographical settings; examine how to integrate rapid research into existing disaster and emergency response structures; identify critical research needs and priorities; identify obstacles and barriers to conducting disaster research; examine health, mental health, social and environmental effects across diverse population groups and first responders; explore ethical and sensitive approaches to conducting disaster research with vulnerable communities; implement and report findings from rapid interventions; explore innovative methods and approaches for both rapid and longer-term research; and support evidence-informed policy decisions for government, private and non-profit sectors post-disaster.

McFarlane and Norris define disasters as “a potentially traumatic event that is collectively experienced, has an acute onset, is time-delimited… [and] may be attributed to natural, technological, or human causes” (10). This Research Topic was launched in Summer 2020 with an explicit aim to advance knowledge and scholarship on the health, social and environmental consequences of natural disasters. We were especially focused on rapid and effective interventions to support affected populations, and on identifying best practices for conducting community-based or scientific research on disaster-related health effects. The guest editors of this Research Topic came together with a shared commitment to promoting ethical and sensitive approaches to research and meaningful outcomes in a post-disaster environment. While this Research Topic focused solely on research related to natural disasters, it offers important insights and lessons for academics, government, non-profit and health system leaders conducting rapid research during public health crises, such as a pandemic.

## Conducting responsive and ethical disaster research

The realities of research on disaster or emergency crises are different from other types of empirical academic research, especially in populations suffering greatly. Health and social science researchers in the affected areas are often untrained in disaster research; the community, people, and services to be studied are often in disarray; and participants in the affected areas may be overwhelmed by the high number of research investigations or needs assessments that occur to examine impacts to residents and the environment (11). The issue of over-research is widely acknowledged by researchers generally, but has received little attention in disaster-related health studies (12). Furthermore, the immediate attention paid by researchers to examine the effects of a disaster on a population may interfere with the time required for people to heal or recover from the disaster.

Researcher sensitivity to the vulnerability of the populations affected is also critical. For instance, research participants may be at risk of re-traumatization and/or physical harm from talking about their experience Pazderka, Brown, Agyapong et al.. Also, inadequate recruitment strategies may cause additional burden or stress on participants, such as feelings of being used for research purposes. Being aware of the challenges, obstacles, and difficulties associated with this area of inquiry prior to conducting research after a disaster, and building in contingencies to deal with any additional burden or distress for participants, may facilitate sensitive approaches to research and more productive and beneficial research efforts.

Recent events have also illustrated gaps in planning for, and rapidly executing, scientific research in the context of a crisis. Although timely research of populations affected by disasters has been identified as a priority for disaster preparedness, response, and recovery; several important ethical issues prior to the onset of disasters need to be addressed, such as minimizing risks, promoting benefits to participants, and attending to systemic inequities some population groups experience with access to health services (13). Oulahen, Vogel and Gouett-Hanna emphasize building on existing local expertise by engaging and co-producing knowledge with the people and communities affected by a disaster (6). Establishing strong research relationships grounded in trust and reciprocity can stand in contrast to the urgency and timeliness of rapid research responses (6).

In this Research Topic, we consider some of these ethical issues by drawing on insights gained from rapid research during or immediately following natural disasters. For instance, the article by Fitzpatrick et al. described that many services and programs that were delivered following the 2016 Horse River wildfire in Alberta were limited in scope and resources and did not account for pre-existing inequities in access to health and mental health services among Indigenous residents and communities, which were heightened after the wildfire. Thériault et al. qualitatively examined the experiences of evacuees during the 2016 Horse River wildfire evacuation and highlighted the importance of tailoring recovery responses to the needs of evacuees, providing support over a longer period of time, and building on local expertise.

Furthermore, important information on the health, social and environmental effects from a disaster that needs to be collected during and immediately following these disasters is often missed because of barriers and obstacles to gathering such data, such as length of time taken by institutional ethics review boards (14), lack of knowledge around how best to integrate research into government response and recovery frameworks, and limited time, funding and resources to study longer-term health and mental effects post-disaster of affected populations. Several articles in this Research Topic highlight how mental health effects among adults, children, and youth persist for several years following a disaster Belleville et al., Brown et al.. For instance, Brown et al. found that mental health effects persisted in youth for years following Alberta's Horse River wildfire and highlighted the need for multi-year mental health support programs for youth in post-disaster situations. Yang et al. showed delayed-onset post-traumatic stress disorder (PTSD) symptoms continue to affect earthquake survivors from Wenchun Earthquake in China 10-years later. Moreover, Thériault et al. argue that few studies adopt a longitudinal lens to examine the evolution of the consequences of disasters. Rodney et al. designed a survey to be deployed quickly to capture a broad snapshot of a population's health in response to a bushfire smoke event in Australia. While the survey allowed the researchers to capture experiences from a broad cross-section of the population, they noted a limitation with this method was the ability to capture complex information such as mental health problems and post-traumatic stress, requiring a more detailed examination of social and economic factors and over a longer period. However, the survey provided, as planned, an important baseline for ongoing research on the long-term effects of exposure to bushfires and bushfire smoke.

## Mental health impacts post-disaster among vulnerable populations

As described above, several articles published in the Research Topic examined increased rates of mental health problems, PTSD, among vulnerable and high-risk population groups. Hyde et al. recruited a cohort of mothers who were pregnant or post-partum during Alberta's Horse River wildfire and found that these women experienced clinically significant PTSD Hyde et al.. Brown et al. reported on results of a survey among school-aged children and adolescents following the same wildfire event and observed higher levels of mental health distress among older students, in females compared to male students, and in transgender and gender-non-conforming individuals Brown et al.. Within this same population group, Pazderka, Brown, Agyapong et al. examined the effects of collective trauma and found that youth who experienced prior trauma had higher rates of mental ill-health post-disaster. Moreover, findings from these studies emphasize the importance of addressing the social determinants of health as protective factors for mental health during a disaster. Also, underserved populations such as Indigenous communities are especially vulnerable to experiencing poor mental health because of pre-existing structural and systemic inequities that existed prior to the disaster Fitzpatrick et al.

## Future directions

To facilitate rapid, responsive, and relevant disaster research, we need rapid-learning research systems that integrate researchers, funders, health and social systems, frontline workers, and community partners asking relevant research questions, using innovative research designs and methods, and leveraging rich, longitudinal data across a range of population groups and communities affected. These systems need to be established prior to a disaster and be ready for rapid mobilization. Disasters are important in the policy process because they can open windows of opportunity for elevating issues onto the government agenda (15, 16). It is therefore important to generate rapid, real-time evidence to catalyze opportunities for policy change and effective responses, supported by lessons learned from the disaster event (17).

## Author contributions

SM lead the submission for the Research Topic in Frontiers, provided oversight on editorial process for article submissions, and lead the writing of the editorial. IW and AWHC participated as the assigned editor for articles submitted to the Research Topic and reviewed drafts of the editorial. All authors contributed to the editorial and approved the submitted version.

## Conflict of interest

The authors declare that the research was conducted in the absence of any commercial or financial relationships that could be construed as a potential conflict of interest.

## Publisher's note

All claims expressed in this article are solely those of the authors and do not necessarily represent those of their affiliated organizations, or those of the publisher, the editors and the reviewers. Any product that may be evaluated in this article, or claim that may be made by its manufacturer, is not guaranteed or endorsed by the publisher.
